# Tracking involvement over time: a longitudinal study of experiences among refugee parents involved as public contributors in health research

**DOI:** 10.1080/17482631.2022.2103137

**Published:** 2022-08-10

**Authors:** Elin Lampa, Anna Sarkadi, Fatumo Osman, Ulrik Kihlbom, Georgina Warner

**Affiliations:** a Child Health and Parenting, Department of Public Health and Caring Sciences, Uppsala University, Uppsala, Sweden; b School of Health and Welfare, Dalarna University, Dalarna, Sweden; c Centre for Research Ethics and Bioethics, Department of Public Health and Caring Sciences, Uppsala University, Uppsala, Sweden

**Keywords:** Patient and public involvement, refugees, parents, longitudinal qualitative research, focus groups

## Abstract

**Purpose:**

Patient and public involvement (PPI) is becoming more common in research, but has been problematized for lack of diversity. While PPI literature increasingly focuses on assessment of PPI on research, a focus on the contributors is less common. This study tracked the experiences of involvement among four refugee parents involved as public contributors in a child mental health trial, over three years.

**Methods:**

The study used a longitudinal qualitative design with focus group discussions. Data were analysed using thematic analysis combined with a longitudinal analysis approach.

**Results:**

The refugee parents’ motivations for being involved changed from focusing on individual benefits to societal change. They initially viewed themselves as guests, which transformed into utilizing the group for social support. Time impacted trust-building positively, with continued collaboration strengthening trust. Practical aspects were dominant in the beginning, which shifted over time to allow more focus on research. They identified several learnings they gained from involvement. A discrepancy in how parents and researchers viewed involvement was identified, where parents saw researchers as owners of the research.

**Conclusions:**

To sustain successful PPI collaboration over time, researchers need to prioritize investment in time and resources, in communication, including working with interpreters, and in continued adjustments.

## Introduction

This study explores the experiences of refugee parents involved in a three-year child mental health trial. The rationale for the study is presented, followed by details of the how the refugee parents were recruited to and involved in the trial, and the findings from a prospective longitudinal qualitative analysis of their involvement. Both the methodological approach and the findings are discussed.

Patient and public involvement (PPI), the practice of involving patients or representatives of the public in research concerning their experiences, is increasingly recognized as contributing with essential perspectives in health research (Domecq et al., 2014). The case for PPI can be made through two main arguments. The first, rights-based, argument states that the public have a right to be involved, especially as research is often funded by the public. This relates to the idea that PPI can empower disadvantaged populations. Secondly, a growing body of literature shows that PPI improves the quality, relevance and impact of research. This relates to the understanding that users’ lived experience is valuable expertise, and comparable, but not identical, to the knowledge of researchers. PPI has however been criticized for mainly involving the “usual suspects” as public contributors, i.e., white, well-educated, and often retired contributors. In recent publications, the importance of diversity and representativeness among public contributors has been raised (Chambers et al., 2021; Nimmons et al., 2021: Oliver et al., 2015). Research teams committed to inclusivity and representation admit to struggling to involve contributors with relevant experiences (Nimmons et al., 2021).

Refugee involvement in research is one example of involving public contributors from a seldom-heard group (Doná, 2007; Filler et al., 2021; Gaywood et al., 2020; Strokosch & Osborne, 2016). In a recent review by Filler et al. (2021), several published examples of refugee involvement in health research were presented. Yet, the authors call for improvements in refugee involvement throughout the research process; in obtaining funding, data analysis and scale up, and for addressing barriers to refugee involvement.

The refugee experience and post-migration context have been identified as powerful determinants of health, making refugee involvement essential for research related to this group (Hynie, 2018). For the same reasons, refugee involvement might differ compared to other groups. Refugees in their early years of resettlement go through several simultaneous transitional phases; being in a new country, learning a new language and culture, becoming parents or their children growing and developing (Hynie, 2018). Therefore, change is expected to occur, but refugee contributors’ own insights on how PPI impacted them is a valuable contribution to the field.

Following the surge of PPI in research, came an academic discussion on evaluating public involvement and its impact (Brett et al., 2014; Crocker et al., 2018; Mockford et al., 2012; Staley, 2015; Staniszewska et al., 2008). The last years have provided the research community with a multitude of PPI assessment tools (Boivin et al., 2018). When evaluating PPI, focus has more often been on the impact on the project and not on the contributors (Brett et al., 2014; Ennis & Til, 2013; Russell et al., 2020). Accounts of contributors’ experiences of being involved in research are often collected as one-time reflective interviews (Faulkner & Thompson, 2021; Werner-Seidler & Shaw, 2019). These contain valuable insights, but details of the experiences risk being forgotten or distorted if retrospectively reporting on a longer project due to recall bias (American Psychology Association, n.d.).

### Rationale

PPI in research, especially when involving seldom-heard groups such as the refugee population, is increasingly utilized in health research. Yet, to ascertain that involvement efforts are truly meaningful and inclusive, there is a need to understand how contributors are impacted by their involvement. This qualitative study adds a longitudinal perspective, where users’ experiences are recorded during the full cycle of a research project. This has the potential to capture if and how the refugee contributors’ experiences of their involvement evolve during the research project. This study can contribute to the knowledge on how PPI activities could be tuned to the needs of contributors, to facilitate the PPI process and make it meaningful for both researchers and contributors.

### Aim

To track the experiences and perceived impact of refugee parents during their involvement as public contributors in a three-year child mental health trial.

## Material and methods

This study was designed as a prospective longitudinal qualitative study. Longitudinal qualitative research (LQR) is theoretically derived from life course sociology and often used in the social sciences, but increasingly used in other academic fields such as health research. Compared to traditional qualitative methods, it is better suited to capture and describe social realities as processes playing out over time. LQR is an approach rather than a method, and can be combined with various qualitative analysis methods. As the dimension of time is added to the analysis, it accounts for temporality and change as well as for contextual factors. Commenced at the outset of a project, longitudinal qualitative research has the potential to both identify experiences at different time points during the project, follow the contributors’ trajectories over the course of the project and explore the experienced impact at the end of the project (Cameron et al., 2019; Hermanowicz, 2016; Holland et al., 2006; Neale, 2016; Saldaña, 2003).

### Setting

This study was part of a trial that ran in Sweden for three years, from January 2019 until December 2021, in which four refugee parents were involved as public contributors. The trial was a randomized controlled trial of a community-based group intervention for refugee children and youth, aged eight and above, and experiencing posttraumatic stress symptoms, called Teaching Recovery Techniques (TRT). It aims to increase coping through psychoeducation and practicing techniques to reduce trauma symptoms. In addition to the seven weekly sessions for the children, there are two sessions for the children’s parents, which include psychoeducation and advice on how to support the child (Warner et al., 2020).

### Recruitment of public contributors

During the trial, four refugee parents were involved as public contributors (Warner et al., 2019). The refugee parents were recruited in the autumn 2018, at a public language school for immigrants in Uppsala, Sweden. Two research assistants visited classes to present the project and involvement opportunity, and received help with interpretation to Arabic, Tigrinya and Somali from the teachers. Interested parents were asked to fill in a short application form, which the research team reviewed, aiming to involve contributors whose situation aligned with the intended study participants’ situation, and who showed interest in the topic and a commitment to involvement. After deciding that involving four parents was suitable for balancing the number of parents against other roles in the project, yet still allowing for some diversity, an Arabic-speaking research assistant conducted interviews with four candidates. All four agreed to become public contributors. After losing contact with one parent after the first meeting, a new parent was recruited through another parents’ network.

The four parents who continued their involvement throughout the project are presented in Table I. The refugee parents all had a residence permit, either permanent or limited to 13 months, at the time of recruitment and one or several children between 8 and 14 years. Their educational background ranged from university education to almost no school at all, and one parent was illiterate. They were all fluent in Arabic; however, one parent’s first language was Kurdish. Letters and invitations were communicated in Arabic and later in the project, on request from the parents, in “easy Swedish”. However, in meetings, all contributors agreed that an interpreter should be present. As reimbursement, the refugee parents received an hourly wage equivalent to that of a research assistant, as suggested by INVOLVE: National Institute for Health Research (2016).

**Table I.** The refugee parents involved as public contributors.

Gender	Age	Country of origin	Occupation	Arrived in Sweden
Woman	42y	Kurdistan	Teacher	2015
Woman	44y	Syria	Housewife	2016
Woman	44y	Syria	Housewife	2017
Man	49y	Syria	Driver	2017

### Collaboration process

The refugee parents became involved at the onset of the trial, when funding was acquired. Thus, the aim and project outline were established, but how the trial would be conducted was not decided. They remained involved throughout the trial, and their last point of involvement occurred towards the end of the trial grant period, where the results were interpreted and dissemination strategies were discussed. The researchers, refugee parents and international advisors involved in the project met two or three times per year over the three years, with an English-Arabic interpreter. When the covid-19 pandemic started, meetings were held online. As the researchers anticipated that live language interpretation in large online meetings would be difficult, the refugee parents met with one or two researchers. These meetings were held with a Swedish-Arabic interpreter. The researchers then represented the refugee parents’ perspectives when in discussion with the rest of the team.

### Data collection

**Figure 1.**
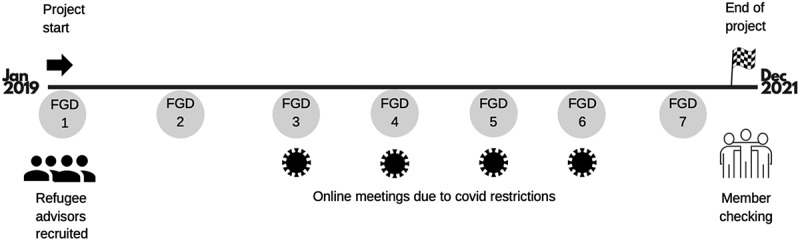
Study timeline.

Data were collected in focus group discussions (FGD), on-site and online (see, Figure 1). After each research meeting with the refugee parents, a FGD was conducted, all using the same interview guide; however, one question was altered to track the experiences of the changing circumstances with online meetings. The FGDs were conducted by two researchers (EL & FO). EL moderated about half of the FGDs, but was present in all; when FO moderated, EL observered. The FGDs were predominantly held in Swedish, with an Arabic interpreter present; although, one of the moderators (FO) sometimes used her Arabic language skills to communicate directly with the participants.

Validation of the language interpretation in the FGDs was conducted, to check for interpreter bias (Liamputtong, 2010). A research assistant, fluent in Swedish and Arabic, with experience of interpretation validation, listened to a randomly selected section of each recording, amounting to around ten percent, as suggested by Wångdahl et al. (2019). She identified issues in two FGDs, mainly regarding the interpreters’ phrasing and that they summarized statements from several participants into one sentence. However, between-participant discussions contributed with higher quality data in the same FGDs. The team decided that the research assistant would transcribe and translate these two FGDs, from Arabic to Swedish, thus making it clear where the interpretation was flawed or biased. The first author transcribed the other FGDs. Both used the guidance from McLellan et al. (2003) and removed identifying details in the data during the process.

### Methods of analysis

The analysis commenced when all data had been collected. In the first step, a thematic analysis of the data was performed, using an inductive approach following the guidance by Braun and Clarke (2006). All authors familiarized with the data and generated initial codes individually. The authors then met repeatedly to generate and review themes.

When preliminary themes were identified, the analysis moved to the second step. Data were organized in a code-based time-ordered data matrix, with *themes* along the Y-axis and *time* along the X-axis (Miles et al., 2019). The purpose was to create an overview of the data, both according to content and chronological order. To understand the patterns of the data and the development of the themes over time, the authors performed an iterative process, moving between the matrix and the raw data (Saldaña, 2003). The analysis process then proceeded with the final steps in thematic analysis, which involved defining and naming the themes as well as writing about the findings (Braun & Clarke, 2006).

Member checking was conducted when the themes were formed, but still undergoing revision, and the authors had just started the longitudinal step of the analysis. This was conducted with synthesized member checking (SMC), a structured version of the commonly used, but less defined, practice of member checking in qualitative research (Birt et al., 2016). While the original SMC method suggests sending a written report, the first author met with the refugee parents, as the researchers experienced that verbal information suited this group better. The first author presented a synthesized version of the preliminary findings and invited the parents to reflect on these in relation to their experiences of involvement. Their reflections were included in the analytic discussion among the authors.

### Ethical considerations

Ethical clearance from the Swedish Ethical Review Authority was sought and approved (ID nr 2018–382). As suggested by Mackenzie et al. (2007), special attention was given to the consent procedure given the complexity of involving refugees in research. They argue that consent procedures might need adjustments for a genuinely informed consent, for example, to ensure that participants do not feel compelled to participate out of fear that it would affect their asylum process or health care (Mackenzie et al., 2007; Mollard et al., 2020). In the present study, consent was recorded orally. A key reason was that one parent was illiterate. A prepared statement was read in Arabic aloud to the refugee parents, and they were asked to state whether they wanted to proceed. The statement included the research intentions and the nature of the questions in the FGDs; that the research findings would be written and published; that their names would not be used in publications; and that the FGD would be audio‐recorded and safely stored. All refugee parents agreed to participate in all FGDs.

The refugee parents were not only research participants, but working in the project together with the researchers. Given the similarity of the roles, the researchers emphasized the difference before each meeting and FGD, allowing the consent procedure to be continuous rather than given at just one point (Gaywood et al., 2020; Holland et al., 2006; Mackenzie et al., 2007). However, there is fine balance between building rapport with research participants and building a friendship. This was exacerbated through the continuous contact in longitudinal research, especially since this often included finding commonalities outside research, such as parenting or mutual interests. The process, and particularly ending the study, had to be communicated sensitively (Cameron et al., 2019; Holland et al., 2006). For the authors, acknowledging that different relationships can co-exist was one part of this balance, together with regular peer reflections to keep the data analysis separate from the more personal relations. However, as these relationships formed part of the process, through the collaboration, there was no clear border.

## Results

In this section, the results are presented: six longitudinal themes, tracking the experiences of the refugee parent over the course of the project (see, Table II).

**Table II.** Themes.

Theme 1	From individual benefits to societal change
Theme 2	Establishing a social network—from guests to peer supporters
Theme 3	Building trust takes time—from polite to candid discussions
Theme 4	Laying the groundwork—overcoming practical aspects to enable involvement
Theme 5	Learning from each other and growing into involvement
Theme 6	We share our experiences—researchers decide what is useful

### Theme 1: from individual benefits to societal change

The first theme tracked the motivation of the refugee parents throughout the three years of involvement. Initially, the parents expressed more individualistic motivations, focusing on themselves and their families. Being motivated by receiving support or attaining benefits for themselves was common, which could for example, be expressed through a desire to receive support for their own health problems.

*So, I wanted it for my mental wellbeing to improve, and to see what can improve my mental wellbeing.*

(FGD 1)

Other parents expressed their early motivations in other ways, such as a general wish to take the opportunity to be involved in anything new that they could benefit from in the future. The parents described they were motivated to becoming involved as they wanted to take all available opportunities to develop skills or establish contacts which could be of use for them or their families. In the last FGD, the parents looked back to the beginning of their involvement, and their first meeting with one of the research assistants, and were able to voice their motivations retrospectively, in a more elaborate way.

*When I met them [researchers] the first time (…) I had been in Sweden for two and a half years, two and a half to three. When she [research assistant] told us I felt encouraged, I signed my name immediately. I wanted to tell someone what happened to me, what is happening to me. I wanted to cling to her so she could help me.*

(FGD 7)

These descriptions of potential individual benefits from involvement were often vague. The parents rarely gave examples of what kinds of benefits they were expecting from involvement, which can be understood as a wish to act on all opportunities to interact with the new society. “For my children” persisted as a common motivation throughout the FGDs, up until the last one where this was not mentioned. In the earlier FGDs the parents described this in a generic way and expressed a hope that taking this involvement opportunity would somehow be of help to their children.

*My motivation is my children.*

(FGD 1)

The concept of mutual gain was also brought up often in the discussions. The parents described that as they contributed to the project and simultaneously gained something themselves, there was mutual gain coming out of their involvement for both them and the researchers, showing a more altruistic side of their motivations.

In the later stages of the project, the discourse around motivations for being involved had changed towards focusing more on changing the situation for refugees in Sweden. As the project progressed, the parents were increasingly hoping that their contribution would have real, tangible impact on the lives of refugees. The idea of changing the situation was based on the understanding that refugees in Sweden were not given a fair chance to integrate and find their place in the society. This was exemplified with the many difficulties the parents had faced in their everyday lives, such as difficulties to find a place to live, to secure a job, their children’s struggles in school and their lack of trust in social services and authorities in general.

*Parent 1:* *We believe there are-*

*Parent 2:* *There are results-*

*Parent 1:* *There is hope.*

*Parent 2:* *There are results, for-*

*Parent 1:* *-for the coming generation.*

*Parent 3:* *That our voices will be heard by people with power.*

*Parent 1:* *Because there are many wars, and people will come, it will not end.*

(FGD 7)

### Theme 2: establishing a social network—from guests to peer supporters

Throughout the FGDs, the social aspect was highlighted as important for the parents’ experiences of their involvement in research. In the first focus groups, what the parents valued mostly was being personally invited to the university facilities, and treated as guests. Practical matters, such being served food, taking a group picture and given an arm to lean on during a walk outdoors, were described as making the parents feel as they were valued guests of the researchers.

*For me, I felt happy, she [research assistant] helped me to close my coat, and she here helped me to walk in the snow (…) Yes, and the one who is responsible got me medicine, and took care of me (…) That’s a luxury.*

(FGD 1)

Being listened to by the researchers was also mentioned in all FGDs, throughout the trial. The parents felt that they were listened to when they were encouraged to elaborate on something they said, when researchers took notes when they talked or when they received the feedback that something they said was important for the project. In the first focus group, feeling listened to or valued was described by one of the parents as something he not often experienced in his everyday life, as he described facing discrimination in the Swedish society. Similarly, one of the mothers later described appreciation of being asked about her experiences and being able to talk about them, as she did not talk about these issues at home.

*Parent 1:* *Yes, they ask us about what pains us.*

*Parent 2:* *Because our husbands do not tell us, do not ask us about what torments us.*

(FGD 7)

The experiences recorded in the first two focus groups focused mainly on the parents’ perceptions of the relationship building efforts of the researchers. As the trial progressed and the parents got to know each other and the researchers, the parents’ focus shifted to a position of mutual interaction. From the third focus group on, the positive experiences of meeting with and sharing issues with other parents appeared to be the most appreciated aspect of the meetings. The parents started mentioning that they looked forward to the meetings, as they had struggled with a parenting issue that they wanted to talk about and get advice on. At this point, the parents had established a social network where peer support around being a refugee parent in Sweden was the core ingredient. In this new network, they shared problems regarding their children, received advice and support from each other and asked the researchers about problems relating to Swedish authorities.

*What’s good is that we get to hear about others’ problems. For example, I have problems with housing right now, getting a place to live. They have problems with residence permit, she with citizenship. They have problems with their children, for example. We get to hear things through the discussion. It helps us to think broader.*

(FGD 7)

An issue arising in the third FGD, ongoing until the second last one, was the fact that meetings were held online, due to the covid-19 pandemic. Even though they were supportive of the decision to meet online, the parents said that the social benefits and the possibilities of getting to know each other were limited by the online format. Considering that the social aspect was such an important part of the meetings for the parents, physical meetings were preferable, yet online meetings were better than not meeting at all.

*I think the difference is that when we met physically, we had livelier discussions (…) before, when we met, we could ask about the private life and have some, talk about other things.*

(FGD 5)

### Theme 3: building trust takes time—from polite to candid discussions

The parents placed emphasis on trust as a cornerstone of involvement, which they connected to sharing personal experiences of value to the project. Thus, trust in both researchers, the research system and the other parents was essential. The discussion around trust and how trust developed was initiated by the parents.

At the beginning of the project, the responses indicated that the parents were eager to please. Their answers were short, with no elaborations, and almost exclusively positive and appreciative of the meetings. They brought up no issues with the meetings, just benefits. Rather than talking about trust—or lack of trust—directly, they responded to questions about their experiences of the meetings with positive remarks about being able to share openly what was on their minds. In the second FGD, one parent described that she and her daughter felt nervous and stressed before the meeting, as they did not know what to expect. However, as she perceived the atmosphere as warm and open, those feelings vanished.

*What made the change is the way how everyone was smiling and everyone was very positive, and whenever we had questions, they were answered with a smile directly in a very welcoming way, I was very stressed that it would not go as smoothly but I felt welcome so that released all the tension and stress.*

(FGD 2)

About halfway through the project, the parents started reflecting about the concept of trust in the FGDs, connecting it to their willingness to share experiences and opinions in the meetings. One parent stated that building trust takes time. This was described as a slow process where researchers needed to be patient, and the parents started sharing when they felt that a trusting relationship had been established.

At about the same time point, one parent also expressed worry about saying things that the researchers would not like hearing. At this point in the project, a major change in the research focus was decided on, which followed on a period of problem solving and discussion around different options, which some of the parents had problematized. This worry of saying things that would upset the researchers was later echoed by other parents, but phrased as apologies for talking to much in meetings.

*Sometimes, perhaps, we say too much, will this upset you, that is what I am scared and worried about.*

(FGD 5)

In the last FGD, the parents shared more in-depth information about their thoughts and concerns in those first discussions three years earlier. They described how they were initially uncomfortable with the researchers talking to their children and therefore asked to sit in on the conversations with the children. They compared this to their views by the end of the project, when allowing the researchers to talk to their children would not feel like an issue—rather they encouraged the researchers to involve their children more. Their initial hesitations were connected both to uncertainty about what their role was in the project, but also to fear (which is elaborated on below). As they themselves reflected, trust had not been established at that point and they did not feel able to share all their thoughts after the first meeting.

As time passed, the parents’ trust in researchers and the research process increased. Parts of this was related to understanding the aim of the project and their role in the group. As things became clearer to them, trust increased. Although, in this regard the parents had different experiences—some found it perfectly clear from start, while others needed more and adapted information to get there. The parents suggested meeting more often would result in an accelerated trust building process. They also suggested that the involvement should continue over a longer period of time, as the trust building process was ongoing but not yet completed. By the end of the three-year project, one parent stated the following.

*And it should not be three years, it should be five, since the longer we’ve been here, the more we learn, more experience, more thoughts. Since, this is important, we’re not yet fully safe, deep down.*

(FGD 7)

In the last FGD, when the parents looked back to their time in the project, they initiated a discussion about fear and worries. The parents talked about a fear of authorities, which they perceived as very common in their social networks, mainly among refugees, where newly arrived refugees were advised to avoid contact with authorities. The parents described that this fear had arisen from living in countries where people were arbitrarily imprisoned, making contacts with authorities a risk for the individual. However, they also described personal experiences, as well as stories told by other refugees, about Swedish authorities, often about social services taking refugee children away from their parents. Initially, this had prevented them from talking freely in the meetings, as the researchers represented the authorities. They expressed fear that what they shared in the meetings would reach other authorities, affecting their lives in negative ways.

*Parent 1:* *Many times, fear.*

*Moderator:* *Tell me, tell me what was it?*

*Parent 1:* *Since we, in the Arab world, are afraid of the police.*

*Parent 2:* *Social services, the authorities (laughs).*

*Parent 1:* *And when we come here, we hear about social services, first time we have heard about it. I have never heard about anything like the social services, that can take your children. That is the reason we don’t want to talk so much.*

*Moderator:* *In this meeting?*

*Parent 1:* *Yes.*

*Moderator:* *What were you afraid of?*

*Parent 1:* *Saying something, and the next day ending up in Syria, or in prison.*

*Parent 3:* *That the social service would hear (laughs) the intelligence service (laughs).*

(FGD 7)

Even though these fears were still present in their contacts with other government representatives, such as social services, they generally trusted authorities more by the end of the project than they had at the start of the project. However, in the context of their involvement in research, the parents talked about these fears as being a thing of the past. Their initial fears that what they said in the meetings would be forwarded to other authorities, were no longer present by the last meeting. This was clear as they were then able to talk freely about their past fears, and clearly outline that careful information, time and feeling welcomed were important components of building trust. However, what was most commonly mentioned as essential for trust was getting to know the researchers personally. They most commonly referred to the assigned contact researcher (EL), who hosted most of the meetings and coordinated the contact between meetings, but also several other researchers on the team who they met regularly. In their descriptions of what changed over time, the researchers seemed to have gone from being representatives of the government, and thus not trusted, to people who they knew and trusted.

*Moderator:* *What changed, from the beginning until now, what has changed?*

*Parent:* *What happened is that we have gotten to know each other.*

(FGD 7)

### Theme 4: laying the groundwork—overcoming practical aspects to enable involvement

An important aspect of the FGDs was the parents’ feedback to the researchers about the meeting, which the researchers continuously incorporated into the planning of the following meeting. That makes this theme distinctly different from the other themes, as the change over time was not just associated to changes in the involvement process, group dynamics or research process, but as a result of deliberate changes, suggested by the parents and incorporated by the researchers. The parents’ feedback changed from, in the early phase of the project, focusing on practical challenges, to—as these practical challenges were solved—moving towards more intellectual, research-focused challenges.

In the first two FGDs, the parents talked about the practical challenges they faced when coming to the project meetings. They described general challenges such as finding their way in a new part of the city and identifying the right bus stop, which they found difficult since none of them spoke Swedish. Another point of discussion was agreeing on the best way to reimburse parents financially, where the team encountered a number of practical issues. The parents were also uncertain whether they would be available for meetings. At the time, the parents all participated in Swedish language education, and their presence was mandatory to receive social benefits. This made the parents uncertain regarding their rights to miss out on some days to instead be involved in research. By the second meeting, the researchers had arranged for travel guidance in Arabic, and a letter to the language school ensuring that the parents worked for the university on certain days, which the parents found helpful.

*I was afraid that if I told them, they would say it was not allowed.*

(FGD 1)

The circumstances for the meetings changed before the third meeting. As the covid-19 pandemic spread to Sweden, the meetings were restructured into smaller meetings online, which caused some new practical challenges to arise. The communication efforts around the practical setup for online meeting were good preparations, according to the parents, but the practicalities of accessing an online meeting for the first time were still challenging.

As the initial practical challenges were solved, the focus of the suggestions from the parents started to shift, towards more research-focused suggestions. In this phase, the parents’ suggestions centred around the meetings such as how to structure the meetings and around research in general, e.g., new topics to research in the refugee field. Most importantly, the parents gave suggestions for the research project, e.g., suggesting that children should be more involved in the projects and identifying potential issues with delivery of the intervention.

The parents also expressed that researchers working with PPI need to understand the practical challenges for the group they involve, as they are likely not the same as for a researcher. The focus on practical challenges in the beginning of this project, such as finding the right bus stop and solving how to reimburse the parents in a way that functioned for them, were important challenges to solve to continue the involvement process. However, the background to these challenges were explained in more detail in the last FGD, where the parents discussed whether it’s appropriate and meaningful to involve newly arrived refugees or not. The parents described life as a newly arrived refugee as chaotic and confusing, where every aspect of the daily life needs to be solved without the knowledge of how to solve it and often with no one to ask. Things that might seem simple to others, were perceived as challenges for the parents during their first years in Sweden.

*Parent 1:* *When I first came here, I had been here for four months, and my daughter suffered an injury, I did not know what to do, where to go, I needed someone to help me, to take my hand, show me the laws, my rights (…)*

*Parent 2:* *That’s just it, when you are new you know nothing.*

(FGD 7)

These circumstances are quite unique for the refugee group, and the parents challenged the idea of how and if newly arrived refugees can contribute to research, with the limited knowledge that they have of the Swedish society. There were mixed ideas about this among the parents, but some suggested that newly arrived refugees should not get involved in research until they have lived in the new country for a year or two, since they would not know enough about the society. Other argued that if refugees received accessible information, involvement early after arrival would not be a problem. However, all agreed that the chaos which was typical for the first time in a new country, could make it more difficult for refugees to be involved in research, and that this need to be taken into account by researchers.

### Theme 5: learning from each other and growing into involvement

This discussion about learning from involvement was initiated by the parents, as they repeatedly mentioned learning new things from being involved in research. Being new in the Swedish society, the parents connected learning to increased possibilities of advancing their situation.

Initially, the parents talked about learning about their children and their children’s health, in order to be the best possible parents, but did not give examples of what they had learnt. However, in the third FGD, a few specific learnings came up: learning about online meetings, which several parents found challenging but managed to solve, and learning some Swedish. One of the mothers had experienced that since the pandemic started her knowledge of Swedish had decreased, as her language education was online and difficult for her to follow. Therefore, she appreciated learning some Swedish from listening to the researcher and then to the interpreter.

*Now I learnt some Swedish as well, when you talked I understood at least a few words.*

(FGD 3)

In the fourth focus group, the first mentioning of peer learning came up, and this became more and more important throughout the following focus groups. The parents learnt about parenting challenges and strategies to solve them, through listening to each other describing their situations and solutions. As an example, the mother with the youngest children stated that she learnt about parenting teenagers, by listening to the other parents with older children.

In the fifth FGD, the parents described that they had learnt more about the Swedish society and that their research involvement had contributed to that, given these issues were discussed during the FGDs on the parents’ initiatives. In addition, by the fifth focus group, the parents also stated that they have become more confident in their role as PPI contributors. They felt more secure about what was expected of them during the meetings, and in their role in general. Finally, being listened to and having their opinions valued was connected to a feeling of hope.

*It raised our confidence, we who come to this society and do not know the language or the traditions, that someone listens to what we think (…) It makes us think our children might have a future in this country, that’s what we want to give them. How we were thinking two years ago, we don’t think the same way now, two years later.*

(FGD 5)

### Theme 6: we share our experiences—researchers decide what is useful

When the parents discussed their contributions to the research project, they exclusively talked about sharing their experiences and their opinions. Throughout the FGDs, the parents said that they felt they could participate actively in the discussion, respond freely to the questions and that they felt listened to when sharing their opinions and experiences. However, they also mentioned that which experiences they shared, depended on which questions they were asked by the researchers.

*Parent 1:* *There was nothing we did not talk about.*

*Parent 2:* *We talked about everything.(laughter)*

*Parent 2:* *It depended on the questions you asked, of course. Then we responded to them as we liked.*

(FGD 1)

Thereby, the parents framed their involvement as passive; they were not in charge over how they contributed to the project or to which topics were raised in the meetings. They talked about research as being the researchers’ own process, of which they did not claim ownership or control, and this understanding remained the same throughout the project. The parents were, however, convinced that their input was important and that the “real world perspective” was of value in research.

When asked whether they thought that their input had an impact on the research, the parents consistently referred back to the researchers. They occasionally expressed that they thought their input was relevant for the project, especially from the third meeting and onwards. However, according to the parents, only researchers can tell what is of value and actually useful for the research project. The parents’ responsibilities, they argued, ended when sharing their experiences and opinions with the researchers. No change in this reasoning was identified throughout the project.

*I think that when we have discussed, you listen to what we think, you consider our thoughts and what we think about things, and then you use what is important in your research.*

(FGD 5)

The researchers’ role in relation to the parents was, according to them, to bring their voices forward. This was something that came up in the late stages of the project, when the parents increasingly brought up the outcome of the project and its potential effect on society, and more specifically on societal support for refugee families. This led them to ask about dissemination of findings and share their hopes for change. In this agenda, they hoped that the researchers would be able to bring their ideas and experiences to those in charge of these societal changes, such as politicians.

When directly asked, the parents did think that they contributed to research, but did not base this on seeing their actual contribution taking place in the research process. Rather, they rationalized that they would not be invited back if they didn’t make themselves useful, or mentioned feeling listened to in the meetings and valued as individuals. Most prominently, however, the parents feeling that they contributed was directly connected to researcher feedback, identifying the changes made in the project based on their input.

*Parent:* *From last time we know now from the results that you presented, that there was an effect of our participation last time, but from this time we will not know until next time.*

*Moderator:* *So what do you think? Do you feel like we have listened to your ideas and will use them?*

*Parent:* *Perhaps (laughs).*

(FGD 2)

## Methodological discussion

### Strengths and limitations

This study has a number of strengths: a longitudinal design, seemingly the first of its kind, following the same group and its PPI contributors representing voices often unheard. Longitudinal data collection can be challenging, especially when involving refugees who might, for voluntary or involuntary reasons, change locations or contact information frequently. In this study, the same individuals, except for one person, were involved in the project throughout the three years. Another strength was that member checking, using the SMC method, was conducted, which was deemed accessible and appropriate for the refugee parents. Through this, the authors ensured that the participants’ views on the preliminary findings aligned with theirs, as well as received additional perspectives. This likely reduced researcher bias, such as confirmation bias (Birt et al., 2016).

One limitation was that all refugee parents were Arabic-speaking and originating from the same geographical region. However, they contributed with diversity regarding education, ethnicity and gender. Due to changes of interpretation agencies and difficulties to find English-speaking interpreters, different interpreters were employed. This raised a concern about interpreter bias. Therefore, a rigorous validation of the translation was performed, which alleviated concerns regarding the participant-to-researcher interpretations.

### Methodological considerations in longitudinal qualitative data collections and analysis

Longitudinal qualitative research requires flexibility, as the intended plan at the outset might not function throughout (Hermanowicz, 2016). During the study, the covid-19 pandemic forced meetings online, and to capture this experience the FGD guide was adapted. During data collection, caution was taken not to let the same person who conducted the meeting moderate the following FGD. However, when another researcher moderated the FGD, the first author observed them to continuously familiarize with the data. This might have hindered the parents from expressing negative opinions, but might also have increased trust. Among the analysts, both researchers familiar with the project, as well as researchers approaching the data with fresh eyes, were represented, which is considered a strength.

When undertaking this study, the authors identified a lack of comprehensive guidance for longitudinal qualitative analysis, specifically for focus group data. This was exacerbated by the study circumstances: the challenges with language interpretation and the changes in PPI activities during the project as well as FGD format. Credibility was ensured through quality assurance of the interview material by checking interpretation and the member-checking procedure after the final FGD. Dependability was enhanced through careful description of the analytical approach and its different phases. In addition, all authors took part in the iterative data analysis process. Transferability to refugee PPI contributors is deemed high given the heterogeneity of participants’ backgrounds together with the specific circumstances of forced migration (Hynie, 2018). However, for the same reasons, transferability to PPI contributors with other types of structural vulnerabilities does not seem appropriate.

## Discussion

This study tracked the experiences and perceived impact of refugee parents involved as public contributors in a child mental health trial. In many regards, the findings in this study align with previous qualitative research on PPI contributors’ experience (Faulkner & Thompson, 2021; Liabo et al., 2020; Thompson et al., 2014; Werner-Seidler & Shaw, 2019). Werner-Seidler and Shaw (2019) reach similar conclusions in their interview study on contributors in mental health research, including motivations for involvement, the social aspects and the need for feedback. Faulkner and Thompson (2021) highlight the emotional work in involvement, adding that ethnic minorities experience increased challenges when being involved in research. These perspectives were not identified in the present study, but might still be factors affecting the refugee parents’ experiences. In Thompson et al. (2014), interviewed public contributors mirror the altruistic motivation the refugee parents expressed in the later FGDs; however, there are few other similarities. The participants in Thompson’s study belong to another demographic group, as they have professional backgrounds and have entered PPI through other civic engagements. The refugee parents in this study implied a goal-oriented mindset when describing their motivations, which can be connected to their ongoing struggles in finding employment and integrating with Swedish-born people, which were topics frequently discussed throughout the FGDs. Finally, the refugee parents considered the social support a core activity, while the researchers rather viewed it as a side effect—albeit a pleasant one. This is a known effect in PPI, as seen in previous research (Faulkner & Thompson, 2021; Liabo et al., 2020).

### Building trust

The longitudinal nature of the findings in this study revealed aspects, relating to change over time, of public involvement which have not before been identified in the literature. One such aspect is the development of trust over time, that the parents established as essential for involvement. This finding is relevant for the involvement of other groups as well, especially seldom-heard groups; however, the enablers and barriers for trust will differ between groups. The refugee parents’ barriers to trust were power-oriented, as it was related to lack of trust in authorities. Trust was established when getting to know the researchers as individuals, which was both expressed by the refugee parents and observed as they increasingly shared experiences and challenged researchers’ ideas. It is likely that this process was delayed due to meeting online. The findings suggest keeping the same contributors involved over longer periods of time as their trust is likely to increase, and their input improve, over time. A key problem is that projects are limited in time, and so is funding for PPI (Filler et al., 2021).

### Aiming for co-production or a mutually satisfactory exchange?

Another important and unexpected finding was that researchers and refugee parents did not seem to have the same idea of what involvement is. This issue raised the question: does this matter? Must all involvement activities aim for fully equal co-production, or can this mutually satisfactory exchange be enough, or even preferable? The answer must be that it depends. One key factor is why the researchers and refugee parents have different ideas. If it was because the researchers failed to provide accessible information about the research process, these issues might have been avoided with training for PPI contributors, and for researchers as well (Staley et al., 2019). Secondly, it could also be related to the refugee parents’ own agency, as they might have chosen the role that best suited them and their current circumstances. In a recent article, Steffensen et al. (2022) identifies several co-existing roles among public contributors in a project, seemingly due to that the contributors pursue different goals and have different abilities, while still contributing to the project. A third factor is the ethical perspective. It might not be unethical to take on different roles in a collaboration, but if the expectations are not clear for contributors from a vulnerable population, it raises ethical concerns about the recruitment process, information sharing and decision-making.

### Is this an example of “good” involvement?

In light of these findings, it is worth reflecting on whether this project can be considered a good example of involvement. Guidelines for meaningful involvement, such as those by Kaisler and Missbach (2019) and Liabo et al. (2020), suggest similar approaches, including continuous involvement, equal partnership, transparency and valuing contributor input. The refugee parents’ experiences in the present study mirror these values to a large extent. In a more specific example, Liabo et al.’s (2020) study identified the involvement practices “informal and welcoming meeting spaces” and “opportunities to share lived experience” as important, which is very similar to what the refugee parents emphasized having appreciated. However, the common understanding of involvement was lacking in the current study. This might not align with “good” involvement and could be a sign of issues with transparency. Although, as Steffensen et al. (2022) points out, it can also relate to that refugee parents simply chose a contributor role that suited them, which is similar to what Kaisler and Missbach (2019) suggest, in giving an opt-in/out option for tasks.

Involvement needs to happen on terms that function for those involved, and preferably this is achieved by the contributors co-producing the PPI activities. The importance of this can be seen in the two themes about laying the groundwork and trust. For refugee contributors, involuntary migration can mean not having the time to learn about the country before you arrive, not being able to bring what you need, and struggling with past traumas as well as worries for friends and family left in the country from which you fled. In addition, grasping laws and society as a newly arrived refugee can be difficult. Time, communication and flexibility in the researchers’ approach towards the public contributors is essential in building a research collaboration that is mutually beneficial.

## Conclusion

This study tracked the experiences and perceived impact of refugee parents involved in a child mental health trial over three years. Through the longitudinal qualitative approach, the study adds a temporal perspective as well as the perspective of a specific group: refugee parents. Both were perspectives lacking in the current literature. When compared to suggestions from the literature on what constitutes as “good involvement”, the parents’ experiences were positive; yet, there were some unexpected findings. The impact of time on trust was clear, with continued collaboration strengthening the trust experienced by public contributors. Whilst practical aspects were dominant at the beginning of the collaboration, these gave way to allow more focus on research activity over time. The last theme identified a discrepancy in how the refugee parents and the researchers viewed the collaboration, where the parents saw the researchers as ultimate owners of the research. This highlights the need for time, communication and flexibility seen in the other themes, as well as better funding opportunities to sustain PPI collaboration over time. These insights are novel and relevant contributions to the PPI literature, and of practical use for all researchers aiming to involve public contributors, especially from seldom-heard groups.
